# Time-Resolved Diffuse Optical Spectroscopy and Imaging Using Solid-State Detectors: Characteristics, Present Status, and Research Challenges

**DOI:** 10.3390/s17092115

**Published:** 2017-09-14

**Authors:** Mrwan Alayed, M. Jamal Deen

**Affiliations:** 1 School of Biomedical Engineering, McMaster University, Hamilton, ON L8S 4L8, Canada; alayedms@mcmaster.ca; 2 National Nanotechnology Center, King Abdul Aziz City for Science and Technology (KACST), Riyadh 11442, Saudi Arabia; 3 Department of Electrical and Computer Engineering, McMaster University, Hamilton, ON L8S 4L8, Canada

**Keywords:** diffuse optical imaging, diffuse optical spectroscopy, functional near-infrared spectroscopy, silicon photomultipliers, single-photon avalanche diode, time-correlated single-photon counting, time of flight, time-resolved spectroscopy

## Abstract

Diffuse optical spectroscopy (DOS) and diffuse optical imaging (DOI) are emerging non-invasive imaging modalities that have wide spread potential applications in many fields, particularly for structural and functional imaging in medicine. In this article, we review time-resolved diffuse optical imaging (TR-DOI) systems using solid-state detectors with a special focus on Single-Photon Avalanche Diodes (SPADs) and Silicon Photomultipliers (SiPMs). These TR-DOI systems can be categorized into two types based on the operation mode of the detector (free-running or time-gated). For the TR-DOI prototypes, the physical concepts, main components, figures-of-merit of detectors, and evaluation parameters are described. The performance of TR-DOI prototypes is evaluated according to the parameters used in common protocols to test DOI systems particularly basic instrumental performance (BIP). In addition, the potential features of SPADs and SiPMs to improve TR-DOI systems and expand their applications in the foreseeable future are discussed. Lastly, research challenges and future developments for TR-DOI are discussed for each component in the prototype separately and also for the entire system.

## 1. Introduction

Diffuse optical spectroscopy (DOS), also known as near infrared spectroscopy (NIRS), is an optical technique to investigate the interaction between light and matter within the optical window of 600 to 1000 nm. In this wavelength range (red and near infrared light), the absorption of water is very low and scattering is the dominant interaction [1,2]. Therefore, incident light in this range can propagate inside highly scattering (turbid) targets up to a few cm until the diffused photons are absorbed or reemitted out of the target [3,4]. The light propagation inside a highly scattering target is mathematically described by a forward problem solver based on the radiative transfer equation (RTE), or its simplified version, the diffusion equation (DE) [5,6]. These re-emitted photons can be collected using photodetectors in either reflectance geometry or transmittance geometry. The reflectance geometry can be used for both thin and thick targets such as newborn and adult heads respectively, but the reachable depth of collected photons is limited to less than 4 cm. The transmittance geometry is only applicable for thin targets (less than 8 cm of thickness) such as breasts and newborn heads [7,8]. Different measurement geometries can be achieved using multiple channels for both sources and detectors, but this is more complex and expensive than a scanning approach that moves around one or more source–detector pairs in order to can the target [9,10]. 

After the reemitted photons are detected, an inverse problem solver is implemented to analyze the raw data of the detected photons from a DOS setup. Then, three-dimensional (3D) or two-dimensional (2D) images of the optical properties of turbid targets are reconstructed. This combination of a TR-DOS system and inverse modeling are what consist of a diffuse optical imaging (DOI) prototype [11,12]. From the reconstructed images, any inclusion or heterogeneity inside the target may be detected, localized, and its size estimated with good spatial resolution (up to few mm) [13,14]. Since the 1970s, DOS has emerged as a powerful technique to explore the chemical properties and compositions of objects in several fields such as agriculture, food, pharmaceutical, and medical imaging [15,16,17]. In the last few decades, the applications of DOI for non-invasive tomographic and topographic imaging of tissues and organs have expanded and are known by several names such as diffuse optical spectroscopy (DOS) [18], optical topography (OT) [19], and diffuse optical tomography (DOT) [20]. In this article, the term DOS is used to describe either the part of TR prototypes that produce raw datasets (DToF histograms) or the prototypes that do not reconstruct images of the targets. On the other hand, the terms DOI and DOT are used to describe any prototype that utilizes an inverse problem solver to reconstruct images from raw data obtained from DOS.

DOT systems can produce 2D or 3D images (slices) in transmittance geometry (detectors and sources are not on the same side) for thin targets (less than 8 cm thickness) such as muscles, breasts, and heads of newborn babies [21,22]. The same can be done in reflectance geometry for thicker or high-absorption tissues, but the depth of the interrogated region in the tissue is shallower than in transmittance geometry [8,23,24]. On the other hand, topographic imaging can be applied in reflectance geometry to reconstruct only 2D images from illumination sources and detectors on the same side [7,8]. DOI systems can be used in optical mammography for tumor detection, localization, and evaluating the response from cancer treatment [25,26,27]. In addition, functional DOI or functional near infrared spectroscopy (fNIRS) is used to take images of the changes in optical properties due to the variation in tissue oxygen saturation (StO_2_) and blood flow in the brain during doing some functional activities [1,21,28].

DOS measurements approaches are categorized into three methods: continuous wave (CW), frequency domain (FD), and time-resolved (TR) [2,21,29]. CW usually employs dozens of sources and detectors which restrict the scalability of the DOI systems and increase the amount data that must be analyzed to reconstruct images [30]. Moreover, CW DOS can only monitor the variation of the optical properties, so without estimating the values of optical properties, its capabilities for structural imaging are limited [7]. FD and TR-DOS can be used to quantify the absolute value of the optical properties. However, with FD-DOS it is challenging to discriminate depths (in reflectance geometry measurements) in comparison with TR-DOS [31]. On the other hand, TR-DOI systems were proven to be the most powerful approach among the three techniques with respect to depth sensitivity and recovery of the absolute value of the optical properties of the targets. TR-DOI has been continuously developed and improved since the late 1980s [32,33]. However, few commercial systems are available to-date (TR-DOT [34]), (TR-DOS [35]), and (TR-DOS [36]). Nonetheless, most of the reported TR prototypes were built in research centers and academic institutions. 

With the use of TR-DOI, many practical limitations—such as large size, high cost, and complexity—were reduced. These improvements are primarily due to the rapid advances in photodetector technology and timing electronics that led to reduced cost and size of photon counting and timing devices by more than three orders of magnitude during the last three decades [37]. Therefore, in this article, we review state of the art TR-DOI prototypes using compact solid state detectors such as Single-Photon Avalanche Diodes (SPADs) and Silicon Photomultipliers (SiPMs). These detectors represent cutting edge technology in the miniaturization and cost reduction of picosecond photon timing and counting applications such as TR-DOI. Therefore, by exploiting solid-state detectors, the usage of TR-DOI is expected to expand in several fields significantly, and more affordable and portable commercial devices are expected within the next few years [37]. To the best of our knowledge, this is the first article to review all reported TR-DOI prototypes using semiconductor detectors and characterize them based on their specific features.

To aid in the logical flow of the recent evolution of TR-DOI prototypes, this paper is organized as follows. In Section 2, the main components of TR-DOI prototypes are described. Next, in Section 3, the parameters which are used to evaluate the performance of the entire TR-DOS systems as well as the figures of merit of the detectors are presented and discussed. In the Section 4, the mechanism, features, and challenges of diffused photon counting for both SiPMs and SPADs are discussed. In the Section 5, TR-DOI prototypes in free-running (FR) mode using either SiPMs or SPADs are described and compared. In Section 6, time-gated (TG) TR-DOI systems, which are only achievable using fast-gated SPAD modules, are discussed. Finally, in the Section 7, the performance of FR vs. TG TR-DOI and the potential usage, features, and possible future developments of TR-DOI using each mode are presented.

## 2. Components of TR-DOI Systems

**Figure 1:**
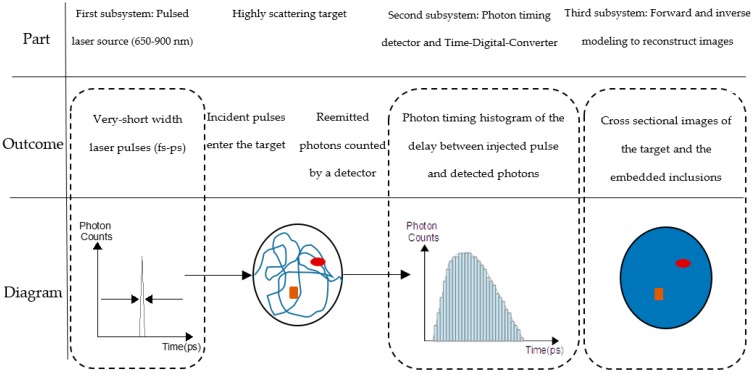
Main components of a typical TR-DOI prototype and the role for each one. From left to right: light illumination, turbid, photon timing, image reconstruction tool.

A typical TR-DOI prototype consists of three main subsystems (Figure 1). The first subsystem is a pulsed laser source(s) that illuminates light into a turbid target. The second subsystem is for photon timing with high temporal resolution (ps range) which is performed by integrating photon counting detector(s) with Time-Correlated Single-Photon Counting (TCSPC) or Time-to-Digital Converters (TDCs) to count photons and record the photon time of arrival (PTA) for each detected photon. TCSPC or TDC produce histograms of the instrument response function (IRF) and the Distribution Time of Flight (DToF) which are measured from the delay between photon arrival time and the injected laser pulse. A third subsystem is required to reconstruct images by using forward and inverse modeling to analyze the histograms from the photon timing subsystems and estimate the optical properties for each point within the target. Eventually, the size, location, shape, and optical properties of a high-absorbent inclusion inside a turbid media, such as tissue, can be investigated based on the variation of the optical properties with respect to surrounding normal tissue [38].

### 2.1. Light Source

The light source should have a very short full width at half maximum (FWHM) (from femtoseconds to hundreds of picoseconds), a center wavelength in the range of 600–950 nm, a narrow spectral wavelength (<±10 nm), and a high repetition rate (tens of MHz). Light sources in this range of wavelengths can be utilized to monitor hemodynamics and estimate the concentrations of oxyhemoglobin (HbO_2_), deoxy-hemoglobin (HHb), total-hemoglobin (tHb), and tissue oxygen saturation (StO_2_). In addition, to monitor water and collagen in a turbid medium, light sources with wavelengths longer than 950 nm can be used [39]. However, the use of silicon semiconductor detectors in prototypes for water and collagen is hindered by the poor performance of the detectors at this longer range of wavelengths (see Section 4). Four types of pulsed lasers are usually used in TR-DOS prototypes: pulsed diode laser [27,40], solid-state laser [33,41], supercontinuum fiber laser [42,43], or the recent low-cost pulsed Vertical-Cavity Surface-Emitting Laser (VCSEL) [44]. Some lab prototypes have used bulky solid-state lasers—such as Mai Tai Spectra Physics—that have higher power and ultrashort pulse width (FWHM in the tens of fs range), whereas other recent setups were developed by using a supercontinuum fiber laser sources (FWHM in ps range) [33]. Also, pulsed diode lasers can only be used with low average power (<2 mW) to maintain a short enough FWHM of the laser pulse (<200 ps) and to avoid increasing the total IRF (*IRF_Total_*) of the system. Therefore, in the literature, it is reported that the pulsed diode lasers maintain low power, which leads to a small contribution from the light source to the *IRF_Total_* of the TR prototype [40,45,46,47]. However, pulsed diode lasers are still widely used because of their affordable cost and availability of many models at different wavelengths from several vendors [48,49,50,51]. Also, VCSELs were used in TR-DOS prototypes and projected to be the most widely used pulsed laser sources for future generation systems [15].

Although the maximum permissible exposure (MPE) was not specified for some internal tissues such as the brain, the power of the illuminated light must be kept lower than the MPE for skin (1.63 W/cm^2^ for a 785-nm source within 1 second integration time) when the illumination source is chosen [52,53]. According to the International Electrotechnical Commission standards (IEC 60825-1:2014), it should be considered that the MPE values vary depending on the exposure duration and the wavelengths used for illumination [52]. In addition, to estimate the MPE for skin during a typical experiment (from 10 s up to 8 h) in the NIR range (700–1050 nm), a simple formula can be used, where MPE (W/cm^2^) = 0.2 × C_4_, and C_4_ = 10^0.002 × (λ−700 nm)^ [52]. Moreover, pulsed lasers with high repetition rates can be approximated as CW laser by using the average power of the pulsed laser which is represented by the energy of the pulse multiplied by the number of pulses in 1 s [54]. The MPE must be considered, particularly for any prototype which is to be used in clinical measurements.

### 2.2. Turbid Targets

The most common method to classify highly scattering targets is based on the similarity of the values of their optical properties. Most tissues such as an adult head are heterogeneous, but some tissues such as breasts and neonatal heads can be modeled as homogenous to simplify the analysis of light propagation [45,55,56]. Turbid targets are categorized according of their type; that is, whether it is a real target such as tissue, or a phantom that mimics the optical properties of a specific organ. Phantoms are more common in preliminary experiments because of their flexibility in shape, size, value of their optical properties and the fact that no permission from ethics boards is required for their use [57]. On the other hand, ethics board approval and patient consent are required for in vivo targets in clinical experiments according to strict protocols.

### 2.3. Photon Counting and Timing

Photon counting and timing that has resolution below the tens of picoseconds range is needed for TR-DOI prototypes, and this can be achieved by two separate categories of equipment. The first being single photon counting detectors integrated with timing electronics such as TDCs or TCSPCs. The second category consists of standalone cameras such as streak cameras or time-gated intensified charge coupled device (ICCD) cameras [33,45]. TR-DOI systems which utilize ICCD or streak cameras suffer from high cost, complexity, and bulkiness that restrict the spread of TR-DOI usage [45]. Therefore, this review focuses on TR-DOI systems of the first category because of improvements achieved in recent years by exploiting the advances of semiconductor detectors to build affordable and compact TR-DOI prototypes.

#### 2.3.1. Photon Counting

**Figure 2:**
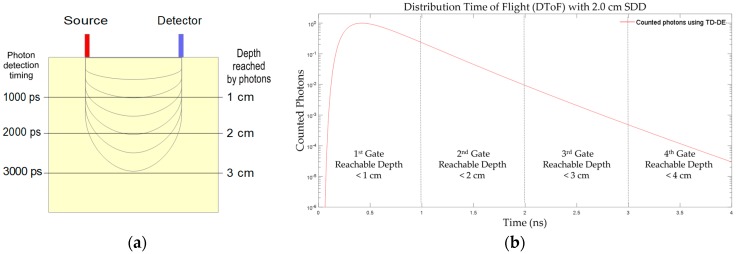
Photons reached areas within the target for each time gate window with different delays. (**a**) An illustration of increasing the reachable depths for photons detected at late gates. (**b**) The positions of gates on the DToF histogram and the possible reachable depth of detected photons during each gate.

For TR-DOS measurements, it is vital to have detection responses faster than 1 ns as well as stable single electron responses for each detected event. This is because having faster detection time helps in better distinguishing between different photon arrival times so that the DToF can have higher resolution. This will help discriminate the differences in the delay between detected photons due to the variation in path-length for each detected photon in the turbid media [7,58]. Several detection technologies can meet these requirements and they were already used in TR-DOI prototypes such as streak cameras [59,60], time-gated ICCD cameras [42,47], photomultiplier tubes (PMTs) [45,61], micro-channel plates (MCP-PMT) [10,62], SPADs [63,64], and very recently SiPMs [65,66]. All the mentioned detectors can operate in the FR and TG modes with the exception of traditional PMTs and SiPMs which work in the FR mode only [43,67]. The benefits of the TG mode are noticeable when the reflectance geometry configuration of measurement as shown in Figure 2. Early photons in the pulse are related to photons that have passed through superficial areas of the target whereas late photons are most likely to have reached deeper areas in the target. These late photons provide useful information [68]. Therefore, the TG-TR approach can be used to achieve null source–detector distance (SDD) in reflectance geometry and produce DToF histograms with higher dynamic range [43]. In Figure 2, an illustration of the reachable depth changes versus delays of detected photons (Figure 2a), and a DToF histogram (Figure 2b) were modeled using a graphics processing unit (GPU) based forward problem solver (MCXLAB), are shown. This was for a homogeneous turbid cube target (4 × 4 × 4 cm^3^) that has a reduced scattering coefficient, absorption coefficient, refractive index of 1 mm^−1^, 0.01 mm^−1^, and 1.37, respectively [69].

An excellent way to distinguish between early and late photons is to use very fast time gating circuits that can record the arrival times of each photon. In the recent years, several types of detectors were used for fast TG-TR-DOI prototypes, such as ICCDs [9,70] and streak cameras [71]. However, these detectors are hindered by the huge numbers of early photons which significantly increase the noise and saturate the detectors [43,67]. Moreover, ICCDs and streak cameras are not compatible with the trend of reducing the cost and minimizing the size of TR-DOI systems. Hence, SPAD detectors are a potential alternative when building fast TG detectors which are capable of ignoring early photons and detecting late photons, within selected delays of picosecond resolution. Therefore, the ultrafast transition time (≈200 ps) of turning the gate on and off is one of the most useful features of TG-SPAD modules because it allows the SPAD to detect only the late photons without being saturated by early photons [43]. This causes the contrast to improve and the number of detected photons within the determined gate-window to increase. Thus, the dynamic range (*DR*) and signal-to-noise ratio (SNR) of the DToF will increase for TG-TR-DOS systems when compared to FR-TR-DOS [43]. The improvement of these factors will lead to significant advances in diffuse optical imaging, particularly in detecting deep inclusions in turbid targets such as tissues. This will in turn lead to improved quality and contrast of reconstructed images. Also, using a close to null SDD allows the maximum level of the lateral spatial resolution to be reached in DOI for highly diffusive targets such as tissues that are dominated by diffusion [43,63,72].

#### 2.3.2. Photon Timing

**Figure 3:**
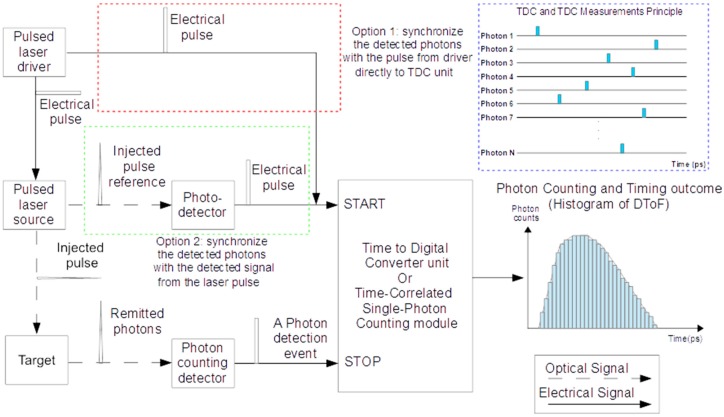
DToF histogram obtained by measuring the delay between laser pulse and PTA. Top-right (in red and green borders) shows two methods (to synchronize the laser pulse with the detected photons. Top-right (blue border) illustrates how the counted photons are stored in the DToF histogram according to the differences in delay for each one of them.

The main output of TR-DOS measurement is a histogram called Distribution Time-of-Flight (DToF) or Temporal Point Spread Function (TPSF) of detected photons, which are generated using TCSPC modules or TDCs [41,73,74]. A DToF is a histogram of the different delay times between the time of triggering of the synchronized injected laser pulse, and the PTA of the detected photons (belonging to the same pulse) at the detector [75]. The width of time-channel should be short (<50 ps) to achieve high temporal resolution [76]. TCSPC modules are superior to TDCs because of their reliability, high maximum count rate of photons (up to 10^7^ s^−1^), and short dead time (<100 ns) [77,78]. However, integrating TDCs with SPAD or SiPM in one chip is a promising approach used in recent TR-DOI prototypes as opposed to using TCSPC modules which are costly and only available commercially [79,80]. To generate a DToF, detectors detect photons that have migrated through turbid targets, whereas for IRF measurements, detectors count photons directly from the source without involving the target. Figure 3 illustrates the principle of TCSPC and TDC measurements, and how all detected photons are stored in the DToF histogram according to the differences in delay between each detected photon and the reference pulse (pulse of laser). The reference pulse can be connected to the TCSPC or TDC from the laser driver (gain switching case) or through the detected signal from a photodetector that measures the injected laser pulse. From the DToF histograms, the optical properties of a target can be determined (see Section 2.4).

### 2.4. Image Reconstruction

**Figure 4:**
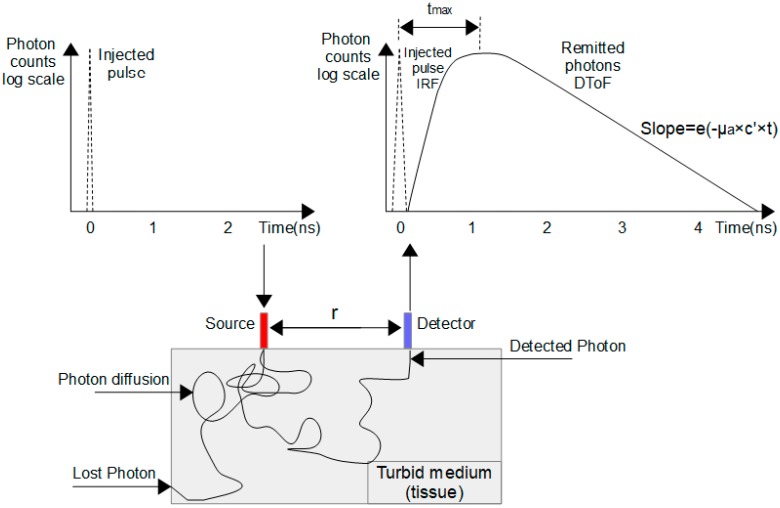
Recovering the optical properties of time-resolved diffuse reflectance measurement for a homogenous target.

Images are reconstructed by estimating the optical properties of each point inside the target. Basically, the optical properties of any object can be recovered by utilizing inverse problem models or some formulas of the analytical solution of the DE that was demonstrated for many geometries [81,82]. For example, in the case of reflectance measurements for a semi-infinite high scattering homogenous target, the optical properties of the target can be recovered by taking information from the logarithmic slope of a DToF’s tail [83,84]. The absorption coefficient can be estimated from the linear regression of the slope of a DToF as follows (Figure 4) [83].

Then, the speed of the light in the medium ($c^{\prime }$), the absorption coefficient (*μ_a_*), time shift between the peak of IRF and DToF (*t_max_*), and distance between the optical fibers of source and detector (*r*) are substituted in the following formula to estimate the reduced scattering coefficient

(1) $$\mu_{s}^{\prime }=\frac{1}{3r^{2}}\left(4\mu_{a}{\left(c^{\prime }t_{max}\right)}^{2}+10c^{\prime }t_{max}\right)-\mu_{a}$$

However, for more complicated geometries and shapes of targets, a regulated inverse problem depends on an iterative forward modeling of multiple DToF of photons detected at different positions are required to estimate the optical properties [8]. 

**Figure 5:**
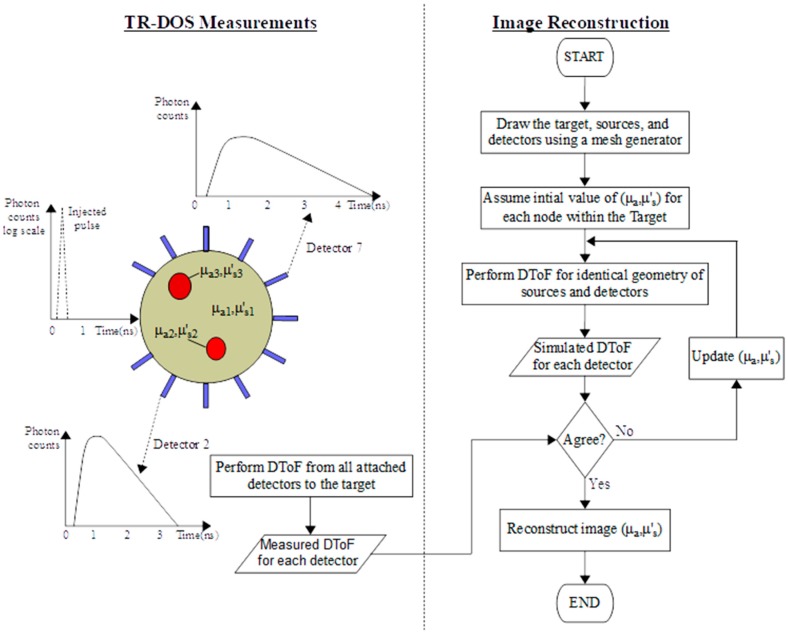
Flow diagram of a TR-DOT prototype, the left and right side represent the measurement setup (DOS) and the flowchart of the inverse modelling respectively.

The DOS prototypes should implement inverse modeling (in OT or DOT prototypes) to recover the optical properties and to detect any inclusions by analyzing simultaneous signals from source–detector pairs attached to a target [10]. For DOT prototypes, Figure 5 represents the iterative processes of the forward modeling until the deviation between measurements and the forward modeling solver for all detectors are reduced enough (comparing the measured data with the forward modeling solver to judge convergence) to reconstruct images for the distribution of the optical properties [8]. The forward modelling simulates the light propagation inside the high scattering target by solving the RTE, or the simplified DE, using stochastic or numerical models such as Monte Carlo (MC) and the finite element method (FEM) respectively [69,85]. To use these iterative processes with all the data points of the DToF are computationally expensive. Therefore, some accelerated FEM approaches were recently demonstrated by analyzing a few critical points from the DToF curves for transmittance geometry measurements (only for simulating data), instead of using all points on the histogram that are not needed, and significantly increases the processing time [86,87].

Generally, high quality image reconstruction requires a prior knowledge of the anatomy of the tissue, but because of the highly scattering nature of the turbid targets, the solution of the inverse problem becomes ill-posed, nonlinear, and ill-conditioned [24,88,89]. Hence, if this anatomical information is considered in the inverse modeling, it is called a soft prior. It is called a hard prior if the anatomical information is also being considered in the forward modeling [8,88]. Overall, many forward and inverse models were developed by several groups to study the light propagation and calculate DToF histograms using different geometries and shapes of targets and estimate the optical properties. However, it is beyond the scope of this paper to discuss these models, although some review papers about modelling and image reconstruction for DOT and OT are recommended for more information [8,19,88,90].

## 3. Performance Parameters of TR-DOI Systems

Several protocols were proposed to evaluate TR-DOI systems such as “Basic Instrumental Performance (BIP)” [91], “MEDPHOT” [92], and “nEUROPt” [93]. BIP is a general protocol that is devoted to evaluating general features of TR-DOS prototypes without considering the sample/target or the role the inverse modeling and image reconstruction subsystem. BIP protocol was successfully applied to TR-DOI prototypes for functional brain imaging [40,94]. On the other hand, “MEDPHOT” and “nEUROPt” are protocols for advanced evaluation which are mainly related to the recovery of the optical properties of a homogenous and heterogeneous target respectively. Therefore, in this paper all mentioned prototypes will be characterized based on their hardware component features and the performance of the whole setup as stated in the publications. Also, it is beyond the scope of this article to include all the parameters from “MEDPHOT” and “nEUROPt” protocols. Most of the parameters that evaluate the reviewed prototypes in this paper will be taken from the BIP and common figures-of-merit (FoMs) for photon timing applications using SPAD and SiPM [95]. These parameters will be described in the following subsections according to the order of the subsystems of TR-DOI prototype, as mentioned in Figure 1.

### 3.1. Light Illumination Properties

Pulsed laser sources should be evaluated according to four parameters; the laser’s average power, spectral width, delivered power, and illuminated area [74]. The laser’s average power represents the generated energy per second. It can be calculated using the formula

(2) $$P_{AVG}=E_{cycle}\times f$$

where *P_AVG_* (W) is the average power of a pulsed laser, *E_cycle_* (J) is the released energy per cycle of a pulse (e.g., length of cycle is 100 ns at 10 MHz repetition rate), *f* is the repetition rate (number of pulses per second (Hz)). A laser beam is emitted in a specific wavelength with a range (e.g., 700 nm ± 5 nm). Good laser sources have narrow spectral width (few ± nm from the center wavelength). The delivered power (*P_Source_*) is the average laser power that is actually delivered to the sample (e.g., consider fiber attenuation). *P_Source_* is the measured output optical power (using optical power meter) from the fiber optics. It can be estimated by subtracting the attenuated power *P_Atten_* from the average power *P_AVG_* of the laser source.

(3) $$P_{Source}=P_{AVG}-P_{Atten}$$

Lastly, the illuminated area *A_Source_* represents the area of the injected light on the surface of the sample. Increasing *A_Source_* leads to a decrease of the spatial resolution for the reconstructed images from the measurement [74].

(4) $$A_{Source}\propto \frac{1}{Spatial\;Resolution}$$

### 3.2. Detection Features

Several features of photon timing in TR-DOI systems should be considered to evaluate the performance of prototype and particularly the detectors. In this paper, we focus on SPAD and SiPM detectors’ characteristics and consider a typical figure-of-merit of photon timing (*FoM_T_*) to summarize the SPAD performance. These criteria are applicable to single pixel SPAD, SPAD array imagers, and SiPM as well, and can be used to compare the detection performance in several application fields. The main parameters are the photon detection efficiency (*PDE*), noise, detection responsivity, dead-time (*T_DEAD_*), timing jitter, fill-factor, and total active area [95,96]. *PDE* is a measure of the ratio of the number of detected photons to the number of incident photons in the active area of the SPAD or SiPM detectors [97,98]

(5) $$PDE=\frac{{\mathrm{Detected}\;}_{ph}}{{\mathrm{incident}\;}_{ph}}$$

The noise in SPADs represents false triggering that may or may not be correlated to time. Dark Count Rate (*DCR*) is the noise that is not correlated with the avalanche process for photon detection [99]. However, after-pulsing (*P_AP_*) and crosstalk refer to subsequent noise pulses that appear after a detected photon is generated. *P_AP_* happens within the same pixel that detected a photon, while crosstalk happens in external pixels [95,99]. The *DCR* ranges between tens to thousands of counts per second for typical SPAD and SiPM detectors [64,100]. The main sources of *DCR* in SPADs are the free-carriers because of the thermal generation that occurs in the depletion region [98,99]. The real *DCR* is extracted from the measured *DCR* using the following formula to eliminate the effects of *T_DEAD_* of the TCSPC or TDC [99,101,102]

(6) $$DCR=\frac{DCR_{M}}{\left(1-DCR_{M}\times T_{DEAD}\right)}$$

Therefore, the total noise in SPAD and SiPM detectors increases with rising temperature [97,98]. The effect of *P_AP_* can be eliminated by applying an adjustable dead time *T_DEAD_* which is adjusted based on an inverse relationship with *P_AP_*

(7) $$T_{DEAD}\propto \frac{1}{P_{AP}}$$

However, long *T_DEAD_* leads to decline the achievable maximum count rate (*Q_MAX_*) of the detector. Most of the parameters stated above have an effect on the value of the *FoM_T_* as [97]

(8) $$FoM_{T}\left(\frac{m}{s}\right)=PDE\times \frac{\sqrt{Active\;Area}}{\sqrt{DCR}}\times \frac{1-P_{AP}}{T_{DEAD}}\times \frac{1}{FWHM}$$

Furthermore, detection responsivity is described by the ratio of detected photons that are transmitted through a slab which has a known *k_p_*(*λ*) transmittance factor.

(9) $$R_{Det}=\frac{N_{Photons}}{T_{Meas}\times k_{p}\left(\lambda \right)\times P_{in}\left(\lambda \right)}$$

where *N_photons_*, *T_Meas_*, and *P_in_*(*λ*) represent the number of detected photons, time of the measurements, and the power delivered to the sample (*P_Source_*) respectively [74].

### 3.3. Photon Timing Histogram

The time width of the channels in the histogram builders such as TCSPC or TDC must be stable and equal [74,80]. To measure if the time widths of the channels are equal, we use the differential nonlinearity ($\varepsilon_{DNL}$). This nonlinearity is estimated using a continuous light source to illuminate the detector, and the TCSPC module to accumulate enough detected photons. These photons are stored in channels based on the differences in the arrival time. Theoretically, the detected photons from a continuous light source should have equal counts for all time channels [74,80]. However, practically, the width of channels of the timing electronics suffers from instability [80]. Therefore, *N_ph_* (number of detected photons) increases with a wider width of the channel (ps), so the $\varepsilon_{DNL}$ can be calculated by comparing the difference between the highest *N_ph,Max_* and lowest *N_ph,Min_* photon counts and normalized by the mean counts using the following formula [74]

(10) $$\varepsilon_{DNL}=\frac{N_{Ph,Max}-N_{Ph,Min}}{\bar{N_{Ph}}}$$

Differential nonlinearity should be measured routinely for the DOS prototypes and $\varepsilon_{DNL}$ should be maintained as low as possible [74].

### 3.4. TR-DOS Prototype

**Figure 6:**
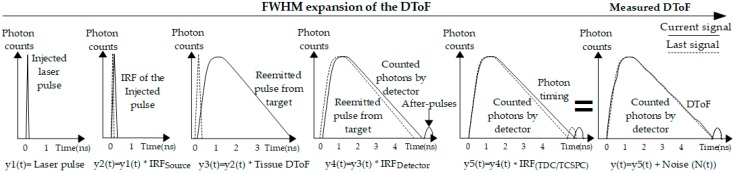
Broaden of measured DToF. Main contributions of the broadening are the time of flight of photons in a turbid medium; noise of the prototype; and the IRF of laser source, detector, and TDC/TCSPC.

TR-DOS prototypes are evaluated based on the raw data produced by the prototype (IRF and DToF) [7,73]. Figure 6 shows how the measured DToF represents the convolution of the *IRF_Total_* and the real DToF (histogram of the reemitted photons from a turbid target), plus the noise in the prototype [74,103].

#### 3.4.1. Instrument Response Function (*IRF_Total_*)

The *IRF_Total_* is the root of sum of squares of the individual IRF resulting from each piece of equipment (except the target) of a TR-DOS prototype, and is given by the equation [104]

(11) $$IRF_{Total}\approx \sqrt{IRF_{laser}^{2}+IRF_{Detector}^{2}+IRF_{OpFb}^{2}+IRF_{TCSPC}^{2}}$$

Having a short and stable *IRF_Total_* is required for all TR-DOS prototypes [105]. The *IRF_Total_* of the setup is estimated from experiments measuring the transmitted light from the laser source to the detector when a thin, highly scattering material with small temporal dispersion, such as a white sheet of paper or a Teflon layer, is inserted between the source and detector [65,106,107]. Using a thin, highly scattering material between laser source and detector ensures that the detected photons are diffused and have multiple directions when they impinge the detector (similar to the re-emitted photons from a turbid target) [106]. From this setup, the *IRF_Total_* can be determined from the FWHM of the measured pulse. All sources of noise, such as *P_AP_* and *DCR*, should be estimated separately for the setup, and they must be reduced to achieve a high dynamic range (*DR*) for the *IRF* and DToF histograms. Moreover, width of *IRF_Total_* needs to be as short as possible because it may distort the accuracy of TR-DOS measurements when it exceeds 1 ns [58,106].

#### 3.4.2. Dynamic Range (DR)

The *DR* represents the ratio of the maximum detectable signal *Sig_Max_* to the minimum *Sig_Min_* detectable signal [95]. High *DR* is desirable for any TR-DOI prototype. *DR* could be calculated using the formula

(12) $$DR=\frac{Sig_{Max}}{Sig_{Min}}$$

Having high orders of magnitude of *DR* is vital for TR-DOI systems, particularly when small SDDs are used in reflectance geometries as they require the system to have at least five orders of magnitude (10^5^) for it to be an appropriate system in applications that require deep detection capabilities such as functional brain imaging [43].

### 3.5. TR-DOI Prototype

High level protocols, such as the “MEDPHOT” [92] and the “nEUROPt” [108], include several parameters to evaluate DOI prototypes according to their ability to detect inclusions in deep regions of a target. These parameters evaluate how good the estimation of the size is, the optical properties and the depth and lateral localization. However, these parameters are not commonly reported in TR-DOI prototypes. Parameters that are typically evaluated are based on the depth penetration, sensitivity, localization, and spatial resolution (depth and lateral) of inclusions. In this review, these features will be used to compare the published performance of prototypes, because there is no standard inverse problem model for image reconstruction using TR-DOI systems [108].

## 4. TR-DOI Using SPAD and SiPM

Single-photon counting detectors are used to generate an electrical signal for each photon that is absorbed. The process of counting incident photons is called the Geiger mode [109,110,111]. PMTs were the most common used detectors in photon counting and timing systems for low light such as TR-DOI [10,16,112,113,114]. The use SPAD detectors have increased recently because they possess several advantages over PMTs: low *DCR*, high quantum efficiency (QE), timing jitter of less than 100 picoseconds, small size, low power dissipation, low supply voltage, high reliability, and ultrafast gating [115].However, there are also some limitations of SPAD detectors: small detection area and low *PDE* in the NIR range [116]. SPADs in standard silicon technology typically have lower sensitivity to photons of wavelengths longer than the visible range (400–700 nm) due to their long absorption depths [117]. Therefore, SPADs in the NIR range suffer from modest *PDE* (less than 30%) since they are mainly fabricated using standard silicon technologies [98,115,118]. However, the *PDE* of SPADs is generally higher than PMTs in the NIR range [118].

### 4.1. SPADs

A SPAD typically consists of a pn junction that is reverse-biased with a voltage that exceeds its breakdown voltage *V_BD_* [98,119]. In Geiger-mode, a large current is created in the depletion layer from one or more free charges (for example, from “dark current” charges or a photogenerated electron-hole pairs) which leads to self-sustaining avalanche multiplication events that increase the current to the milliamp range with sub-ns to ns rise times [97,120]. The avalanche current flows into the junction until the quenching circuit lowers the bias voltage below the *V_BD_*. Biasing of the SPAD is done by waiting for the adjusted *T_DEAD_* (also known as hold-off time) after each pulse to eliminate the *P_AP_* effect. Then, the SPAD becomes ready for triggering again in the Geiger mode by a new photon. The length of *T_DEAD_* varies from ns up to 10 μs, which is important to increase the sensitivity and to maximize the photon counts of the SPAD, particularly in the NIR range [109]. Because of the low absorption coefficient of silicon in the NIR range, the larger thickness of the absorption region plays a significant role in increasing the detection efficiency of the SPAD. However, timing jitter in the detectors increases with thicker absorption regions. Therefore, there is a trade-off in this issue for TR-DOS measurements because they require very short timing jitter (<500 ps) and acceptable *PDE* [109]. Consequently, many of the reported schemes and designs of SPADs focus mainly on reducing the *T_DEAD_*, *IRF_Detector_*, and noise levels such *DCR* and *P_AP_* [95].

#### SPADs Categories

SPAD detectors can be categorized into two types based on their implementation technology: standard silicon (Complementary Metal-Oxide-Semiconductor) CMOS technology and custom silicon technology [98,99]. Several prototypes of SPAD based on CMOS technology were widely implemented by many research groups for variety of visible range applications. In general, SPAD detectors are fabricated in standard CMOS technology based on a thin depletion region (1–2 μm). The main limitations of CMOS SPAD are modest PDEs especially in the NIR range [120,121], worse timing resolution [122], higher *DCR* per unit area [123,124], and more *P_AP_* in comparison with SPADs fabricated using custom silicon technology. The timing resolution (*IRF_SPAD_*) can be as short as 55 ps and 30 ps for CMOS and custom technology, respectively [125,126]. It is worth noting that the timing resolution for SPAD increases to above 100 ps when the diameter of the detection area reaches 100 μm or larger in CMOS, whereas 35 ps time resolution was demonstrated for 200 μm diameter SPAD in custom silicon technology [99,126,127]. 

**Figure 7:**
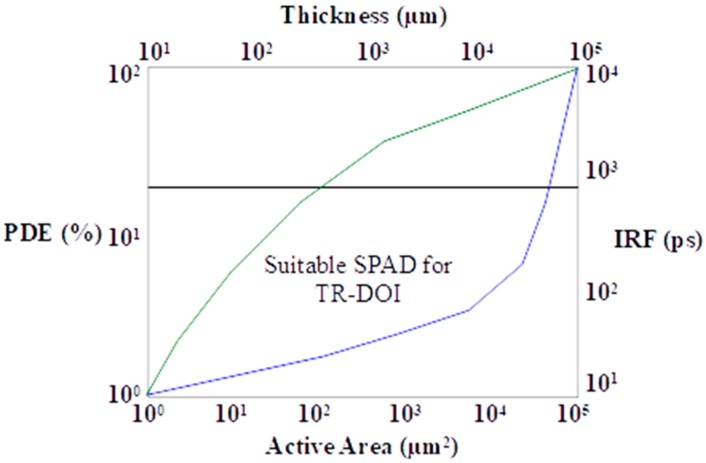
Compromise of SPAD: blue and green curves represent the *IRF* and the *PDE* changes with increasing thickness and the active area respectively, whereas the black line indicates maximum acceptable *IRF_SPAD_*.

Figure 7 shows that it is desirable to enhance the *PDE* without increasing the *IRF_SPAD_* to more than a few hundred ps to be appropriate for picosecond photon timing applications such as TR-DOI. For example, a popular custom silicon technology SPAD SPCM produced by Excelitas Technologies has high *PDE* (>50% in 600 to 800 nm), but it was never used in TR-DOI prototypes because of its large *IRF_SPAD_* as a result of having a thick depletion layer (20–25 mm) [128]. Although custom SPAD technology is a potential approach to optimizing SPAD characteristics and minimizing the limitations, standard CMOS is a superior option for TR-DOI system regarding size, cost, and compactness with having TDCs on the same SPAD chip [80,116].

All reviewed TR-DOS prototypes were implemented with SPADs based on the front-side illumination (FSI), although a backside-illuminated (BSI) CMOS SPAD based prototype was reported as well. 

### 4.2. SiPM

A SiPM is an array of hundreds or thousands of SPADs connected in parallel, and perform as a single large area detector (few mm^2^) with two terminals (one cathode, another anode) [107,129]. The total area of the SiPM can be estimated by multiplying the number of pixels by the fill-factor (FF) for each pixel [95]. When each SPAD connects to one passive quenching circuit it is called an analog SiPM. If an active quenching circuit is connected to each pixel, this is a digital SiPM [95,130]. In analog SiPMs, the number of photons can be estimated from the output current which represents a summation of all photons that are absorbed [95]. However, each single SPAD in digital SiPM is connected to a circuit to generate a signal for each counted photon, and a quenching circuit to turn off the SPAD when it exceeds the maximum time of activity [130,131]. Presently, most commercially available SiPMs cannot reach high enough resolutions to perform single photon counting with high temporal resolution in (100 ps range) without a custom module integrated with the SiPM to extract the timing information for each photon, as demonstrated in [101]. Overall, SiPMs combine the benefits of both photocathode-based (e.g., PMTs) and solid-state detectors such as large active detection area (few mm^2^), affordability, simplicity, compactness, high quantum efficiency in the NIR, low bias voltage, and non-sensitivity to magnetic field. However, SiPMs suffer from low dynamic range of around two orders of magnitude (10^2^) and a long diffusion tail because of the sequence of carriers generated inside the detector for each single photon response [65].

### 4.3. SPADs Operation Modes

**Figure 8:**
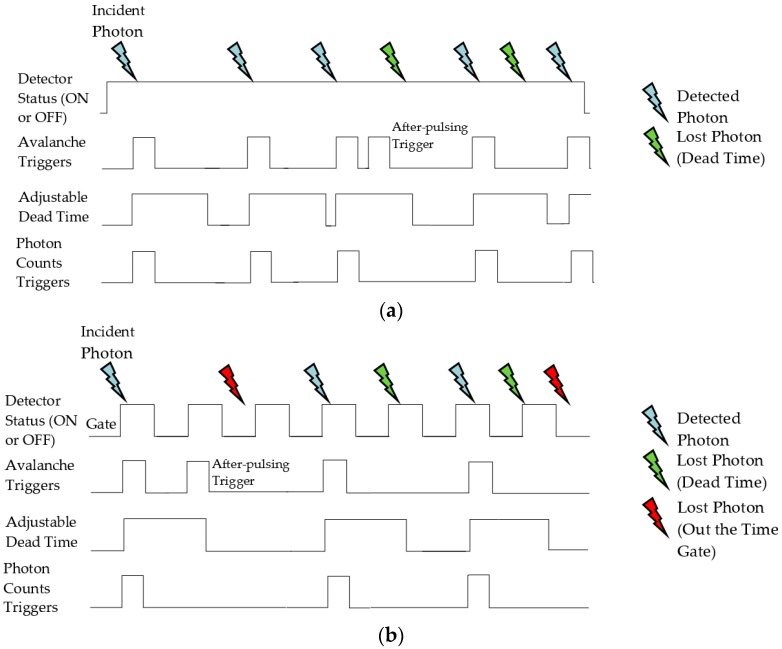
Photon counting process for identical incident photons using (**a**) FR detector, (**b**) TG detector. Reasons of missing photons are indicated on the right for each one.

Detectors can be classified into two categories based on their operation mode: free-running (time-invariant) or time-gated (time-variant) [132]. Free-running SPAD detectors are ready to count any incident photon on the active area, but are kept off only during an adjustable *T_DEAD_* after each photon counting process to reduce the *P_AP_* effect [133,134]. However, TG SPADs are completely blind to the photons impacting the active area when the gate is turned OFF. Photons can only be detected if they arrive while the gate is ON [135]. That is, FR SPADs are always biased above the *V_BD_* to stay ready to count impinging photons, whereas TG SPADs are periodically biased above the *V_BD_* to detect photons only within a very short, precise, and synchronized time gate window [134,135]. Figure 8 illustrates the main differences between photon counting in FR and TG detectors (photons—blue colored symbols), reasons for losing photons (photons—green colored symbols) in FR and (photons—green and red colored symbols) in TG, and the importance of *T_DEAD_* to reduce the effects of false triggering, particularly *P_AP_*.

Later in this section, the major differences between FR and TG TR-DOI prototypes will be described.

### 4.4. Features of the Detectors

Although several TR-DOI prototypes were built using SPADs or SiPMs, seven detectors were actually utilized in the reported prototypes. Five of these detectors were SPADs [116,126,136,137,138], and two were SiPMs [139,140]. Three of these detectors are state-of-the-art (PDM series) SPAD modules with three sizes of active area (diameters 50, 100, 200 μm) fabricated in custom silicon technology by Micro Photon Devices (MPD, Bolzano, Italy) [126,136,137]. Table 1 shows a summary of the features of the detectors. An abbreviated name will be used for each detector to indicate which one is utilized in each reported prototype.

### 4.5. FR vs. TG TR-DOI

**Figure 9:**
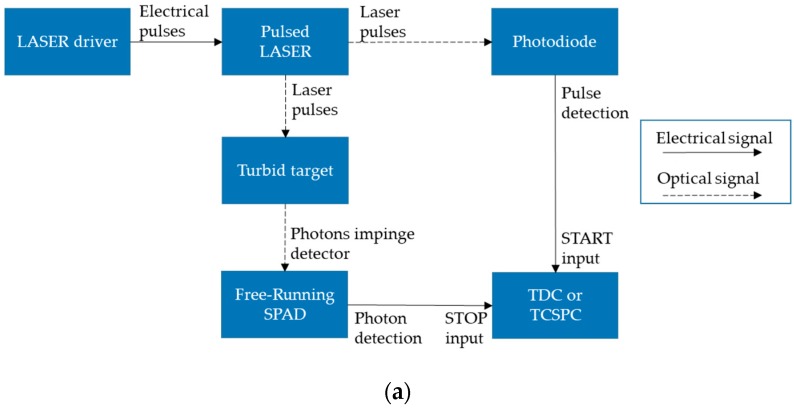

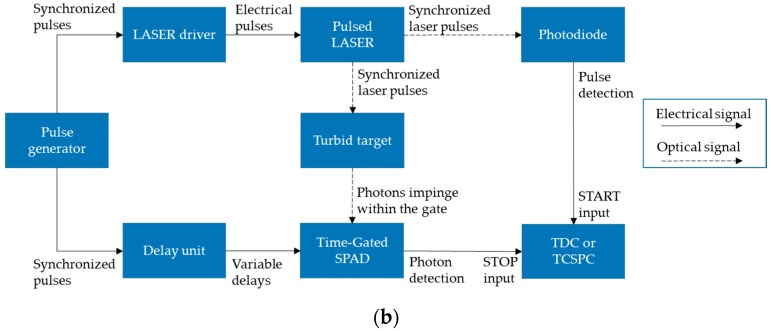
Components of TR-DOS prototypes using (**a**) FR detector, (**b**) TG detector.

On the other hand, the major difference between FR and TG TR-DOI prototypes is the necessity to synchronize the time gate windows with the PTA of the reemitted photons from the target. Therefore, TG-TR-DOI prototypes require a common pulse generation unit to trigger the laser pulses and time gate windows in the same time precisely. In addition, a delay unit is required in TG-TR-DOI prototypes to adjust the delay of the gate and enable the detection to focus only on early or late reemitted photons. Nevertheless, FR-TR-DOI prototypes are simpler than TG because the laser pulse source is triggered from the driver (internally) according to the adjustable repetition rate in the range of one to tens of (MHz). Moreover, the detector is always on and able to count incident photons unless a photon arrives during the *T_DEAD_*. Figure 9 shows the differences of the typical components between FR and TG-TR-DOI prototypes using semiconductor detectors.

## 5. Free-Running TR-DOI

Several FR mode TR-DOI prototypes using SPADs or SiPMs have been reported since 2011 and 2015 respectively. In this section, the FR-TR-DOI prototypes are classified into three categories according to their detectors such as CMOS SPADs, custom SPADs, and SiPMs. Six TR-DOS and DOI prototypes based on CMOS SPADs, custom silicon technology (PDM series) [126], and SiPMs were reported. Each prototype is described briefly, and a summary of the main features of the FR-TR-DOI prototypes is given later in Table 2.

### 5.1. CMOS SPADs

An early non-contact TR-NIR imager (fabricated using standard CMOS HV 0.35 μm) was reported in [138,141]. This SPAD, named CMOS350 in Table 1, consists of 128 × 128 pixels where each row of the pixels are connected serially to 32 TDCs, each of which has a timing resolution of 97 ps. However, it must be noted that there is a compromise between increasing the number of the integrated on-chip TDCs with reducing the fill-factor and *PDE* of the SPAD imagers which degrades the detectors capabilities in very low light intensity applications such as DOI. This setup works in the reflection mode to avoid the restriction of the thickness of targets, and it aims to merge the advantages of the conventional CCD systems with information of PTA. Two beams of light are generated by a line diffuser and a collimator to simplify the image reconstructions that consider the differences in the absorption coefficients μ_a_. Moreover, SDD is kept small (2 cm) to increase the intensity of detected light and improve the SNR. The developed inverse problem modeling analyzes huge amounts of data from the imager and achieves excellent spatial resolution (5 mm at 1 cm depth of inclusions), and good depth sensitivity and quantification of μ_a_. However, the main drawback of this prototype is the long acquisition time (six minutes for each row of pixels) and the small active area of sensor (6% FF of 3.2 × 3.2 mm^2^) in comparison with the sizes of commercial CMOS and CCD sensors [138].

Another TR-DOI imager (called CMOS130 at Table 1) depends on a novel backside-illuminated (BSI) CMOS SPAD was reported in [116]. Two arrays of SPADs (1 × 400) BSI using CMOS 130 nm technology and 3D multi-wafer stacking CMOS process were implemented. Direct Bond Interfaces (DBIs) were used to bond two standard CMOS wafers face-to-face. BSI is an approach that fabricates SPADs with thick multiplication region which is useful in increasing the sensitivity of light in the NIR range. One hundred TDCs were integrated into pixels arrays to calculate the PTA for each detected photon and every eight pixels share a single TDC. Moreover, if more than one photon arrives in one cycle, only one of them will be recorded during the same clock cycle. In this prototype, the *IRF*s of the laser pulse, SPAD, and TDC are 20, 260, and 49.7 ps, respectively. The *IRF_SPAD_* is enhanced significantly (for light detection at 750 nm) from 500 ps down to 260 ps by increasing the excess bias voltage from 1.5 V to 2.25 V [116]. This SPAD imager has Photon Detection Probability (PDP) higher than 12% in the range from 650 nm to 800 nm, but the *DCR* (median 35 kcps) is 10 times higher than the *DCR* of a standalone SPAD in multichannel SiPM implementation using BSI in [142].

### 5.2. Custom SPADs

Recently, a TR-DOI for the imaging of small animals was demonstrated in [58] and two versions of the multichannel prototype were built. The first generation of that prototype was implemented with PMT detectors that have large photosensitive areas (diameter ≈ 8 mm). In the second generation, the PMT detectors were replaced with SPADs that have a small photosensitive area (diameter ≈ 50 μm) named PDM50 in Table 1. The major enhancement the second generation of the prototype was the ability to obtain a much higher temporal resolution where the total *IRF* ≈ 55 ps compared to ≈ 200 ps in the first generation system. The biggest contributor to the temporal resolution enhancement in these prototypes comes from the detectors where the IRF of the SPADs is shorter than 42 ps and the IRF of the solid state pulsed laser is very good (FWHM ≈ 4 ps). In this prototype, 3D images are reconstructed by exploiting time of arrival of early photons (EPTA) which focus on the photons that have the shortest path-length from the source to the detectors. Images were reconstructed slice-by-slice similar to the concept of Computed Tomography (CT). To-date, this TR-DOI prototype has the best temporal resolution among all published FR-TR-DOI systems. It consists of seven SPADs, and each one is coupled to a TCSPC module to generate DToF histograms of cylindrical phantoms and small animals in transmission geometry [58].

### 5.3. SiPM

The validation of SiPMs for TR-DOIs began in 2015 when the parameters of detection performance—such as IRF, *PDE*, *DCR*, and *DR*—for different commercial SiPM models were compared in [129]. 

In [44], a TR-DOS prototype using both SiPM and CMOS SPAD were validated in vivo for functional brain imaging using FR and TG measurements, respectively. This work proved the validity of using large active area SiPM in TR-DOS and the depth sensitivity (3 cm depth) which are comparable with bulky and expensive state-of-the-art TR-DOS systems. Moreover, the first probe using SiPM (SDD 30 mm) had provided deeper depth sensitivity than the second probe (SDD 5 mm) using TG CMOS SPAD (2.5 cm). However, all of them can reach the brain tissue of an adult when they used for functional brain imaging. Moreover, in [107] the probe using SiPM was evaluated using the three abovementioned protocols BIP, MEDPHOT, and nEUROPt. This TR-DOS prototype using SiPM demonstrated high accuracy and linearity in quantifying the optical properties as well as good depth sensitivity for detecting inclusion with acceptable contrast. On the other hand, the prototypes in [44] and [107] are for TR-DOS compact prototypes that used light at 690 nm in the tens of MHz repetition range and 1 mW average power from a low cost and small size VCSEL source [143].

Recently, in [65], the feasibility of using SiPMs for a TR-DOT scanning (reflectance geometry) prototype was evaluated based on several parameters such as depth sensitivity, absolute quantification of the optical properties, size estimation, and both lateral and depth localization. A supercontinuum fiber laser was used to illuminate light at 820 nm (26 ps IRF) into a liquid phantom [144]. The reemitted photons were collected at two points (3 cm SDD) by fibers connected to two SiPMs from Excelitas Technologies, Canada (named SiPM1 in Table 1) with 1 mm^2^ active area for each [145]. Five standard black cylinders of different sizes with high absorption inclusions made of black polyvinyl chloride (PVC) were embedded inside the liquid phantom at different depths (10, 15, 20, 25, 30 mm) [146]. The results of all the parameters were good down to 20 mm depth, but they all degraded for deeper inclusions. It is worth noting that the absolute quantification values of the optical properties of the inclusions are not accurate, mainly when they are embedded deeper than 20 mm, but this is a common limitation of DOI technology when prior knowledge is not used [13].

More recently, a compact (20 × 16 × 5 cm^3^), portable, low power consumption, and low cost TR-DOS system was developed and reported in [66]. This prototype used 2 mW average power pulses at 830 nm and 670 nm wavelengths with up to 40 MHz repetition rates. Reemitted photons were collected using SiPM (SiPM1 in Table 1) with a 1 mm^2^ active area which is connected to a custom made TDC (10 ps resolution) to generate IRF and DToF histograms. Although no images were produced from this prototype, short and stable IRF (<290 ps) were measured, and scattering and absorption coefficients were estimated from the DToF histograms of seven homogenous solid phantoms in reflectance geometry [66].

In [147,148] a TR-DOS prototype was demonstrated for diagnosing locations with bone prominence using two detectors: SiPMs (named SiPM2 in Table 1) and an InGaAs PMT [149]. The absorption and reduced scattering coefficients were estimated (from the raw data of prototype) over a broad range of wavelengths (600–1200) at six locations of bone prominence in the human body. This work proved that seven of the main constituents of tissue such lipid, water, collagen, HbO_2_, HHb, blood flow index (BFI) and StO_2_ can be quantified. Extracting this information about tissue components can lead to diagnosing some bone related pathologies. It should be considered that, in contrast to SiPM, the performance of InGaAs PMT is good at wavelengths greater than 900 nm. Therefore, such PMT detectors can be used when extracting information about constituents of tissue such as collagen and water in this wavelength range [147,149].

Moreover, a commercial TR-NIRS clinical oximeter was reported in [35]. This system uses three diode laser sources (755, 816, and 850 nm wavelengths) to illuminate the light, MPPC SiPMs connected to TDC units are used for photon counting and timing (item details not specified) [150]. Using three wavelengths of laser sources aims to distinguish between HbO_2_, HHb, and tHb and quantify their percentage. Although this prototype suffers from large *IRF_Total_* (1.5 ns) the outcome was similar to TRS-20 bulky commercial prototype [34], and better than spatially-resolved spectroscopy (SRS) commercial prototype (NIRO-200NX) [151].

### 5.4. Comparison of FR-TR-DOI Prototypes

In Table 2, a summary of the main components and the major features of the above-mentioned FR prototypes is provided. Features and details for each component of the FR-TR prototypes are stated. The first part of the table (light source) focuses on parameters of light illumination in these prototypes. The second and third parts indicate features of the photon timing subsystems (detectors and TCSPC/TDC) while the fourth and fifth parts summarize the details for the turbid targets used, and the conditions of the measurements. The sixth part summarizes the method and performance of image reconstruction, and these details are only available for FR-TR-DOI prototypes. 

## 6. Time-Gated SPADs

Several TR-DOI setups based on TG SPADs were reported since 2008. All prototypes used fast-gated SPAD modules (PDM series) with different sizes of active area (see Table 1). 

A very early TG TR-DOS system was reported in [152]. There, they used two pulsed diode lasers (672 and 758 nm (Picoquant, Berlin, Germany)) to illuminate two different targets (solid homogeneous phantom and in vivo head of an adult) [51]. Reemitted photons were collected in reflectance geometry using fibers at two different distances of 2 mm and 20 mm and the photons were transmitted to an SPAD of 50 μm active area (named PDM50) through a 50× microscope objective. Two different widths for the SPAD gates were used (1500 ps and 500 ps) and the rise time was around (800 ps) with delay steps of 500 ps. The *IRF_Total_* was measured for the two laser sources (90 ps for 672 nm source and 220 ps for 758 nm source). This setup proved the benefits of using time gating to improve the spatial resolution and the depth sensitivity. The contrast of detecting deep buried inclusions (18 mm) within a turbid medium for small SDD is better with late time-gate windows. However, the contrast is better with early gates for the larger SDD (20 mm). Finally, the setup of TG mode was applied in-vivo for functional brain monitoring with very good contrast and sensitivity to detect activities in the brain cortex [152].

In [43], the researchers extended their work to investigate the capabilities of the TG versus FR for two SPADs (PDM100 and PDM200 [137]). They proved the advantages of TG to provide a large *DR* which leads to higher spatial resolution and better sensitivity for deep inclusions. In this setup, the gate width for SPADs is flexible from less than 1 ns up to 10 ns by very small steps of 10 ps using an external passive delayer, and the gates can be turned ON within less than 200 ps. The decay time is 85 ps and 240 ps and active area diameter is 100 μm and 200 μm for PDM100 and PDM200, respectively. To compare the signal from TG vs. FR, a proper background subtraction of the noise is needed for the curves to increase the SNR at longer acquisition times. It was concluded that, in reflectance mode of TR-DOS measurements, using TG can achieve up to eight orders of magnitude (10^8^) of *DR* within a measurement time much faster than FR which requires a much longer and impractical measurement time to reach the same *DR* [43]. 

The first in vivo test for functional brain monitoring using both FR and TG SPAD was reported in [63]. There, they performed measurements using a setup containing two laser pulses (710 nm and 820 nm) to distinguish the effect of a finger tapping task on HbO_2_ and HHb dynamics of an adult brain. A fiber for detection was attached at 6 mm from the laser fiber and connected to the MPD-SPAD detector through a band pass filter used to transmit the re-emitted photons. They applied 5 ns gate widths with 700 ps second delay to focus on late photon detection. Their measurements confirmed the advances of the TG over FR in distinguishing brain tasks with better contrast [63]. 

Thereafter, in [135] a Monte Carlo model was developed to simulate the effects of variation of the gates widths on the light propagation and the collected photons for each gate. This model confirms the conclusions made about the *DR* for FR and TG SPADs, as well as the performance of two differently sized (active area) TG-SPADs that were reported in [43]. Therefore, by applying the time-gating with a stable open gate that is synchronized precisely and long enough (e.g., 4 ns) to collect the reemitted photons, the response of the system will be similar to a FR SPAD, but with much higher *DR* [43,135]. It was realized that PDM100 which produce smaller *IRF_Total_* (150 ps) and has shorter decay time (85 ps) can distinguish inclusions up to 3 cm in depth. On the other hand, measurements by PDM200 could gather information only from the early photons that primarily come from superficial regions of the target. The main cause of this limitation is the long decay time (240 ps) and larger *IRF_Total_* (300 ps) [135].

In [153], a non-contact TR-DOS prototype was reported which was used to investigate the contrast variations of inclusions buried in different depths using fast gating windows at null SDD. To illuminate the phantom, 690 nm light with FWHM < 100 ps was used in reflectance geometry with a SPAD detector. A PDM100 SPAD was used, with a fixed gate width (6 ns) and nine measurements with constant delay steps (250 ps) from 0 to 2000 ps were taken. Good SNR was demonstrated for late photon (with delay > 1 ns), and good depth sensitivity (up to 20 mm) with the last gate (2000 ps delay). The contrast was calculated experimentally with different delays and depths of inclusions, and these results agreed with the predictions using a custom Monte Carlo simulator [153]. Eventually, this non-contact TR-DOI prototype was extended and validated successfully for in vivo applications such as functional brain imaging for adults [132,154]. Two illumination wavelengths (760 and 860 nm) were used in the system to distinguish the changes of the HbO_2_ and HHb in healthy adult brain by running the measurements during motor and cognitive tasks that activate the brain [132].

Recently [155], the performance of a non-contact TG-TR-DOI prototype was compared to a clinical fiber-based FR-TR-DOI prototype that uses PMTs named “fOXY” (more details on “fOXY” are provided in [45]). Some parameters from “BIP” and “nEUROPt” protocols—responsivity, temporal resolution, dynamic range, contrast, and spatial resolution—were used to evaluate the two systems. “fOXY” prototype used tens of source detector pairs with 3 cm SDD whereas 5 mm SDD was used with the non-contact prototype. The non-contact TR-DOI prototype had better temporal resolution, *DR*, and spatial resolution. However, “fOXY” had superior responsivity and contrast except for contrast from late photons by the non-contact system. The non-contact prototype had better results for deep inclusion detection which is vital for in vivo applications such as functional brain imaging [155].

Another setup for TG and FR TR-DOT in reflectance mode was integrated [156,157]. The depth sensitivity with a Mellin-Laplace Transform (MLT) algorithm and using PMT detectors was already studied in [41,158]. From this study, it was realized that deeper depth sensitivity required a higher *DR* of the DToF which is only possible by using fast-gated SPAD detectors. The main purpose of this work was to investigate how the accuracy of depth localization and detection of inclusions could be improved. Therefore, measurements using nine gates (5 ns width for each) for two small SDD—5 mm and 15 mm—were made. In this prototype, a pulsed laser at 820 nm with 26 ps FWHM was used to illuminate a liquid phantom with cylindrical solid inclusion (8 mm diameter and 6 cm height) that is embedded at three different depths (13, 19, 25 mm). A TG SPAD, the PDM100 in Table 1 [115] with a variable width for the gate windows (a few hundreds of ps up to 10 ns with 10 ps resolution) was connected to a TCSPC board (SPC-130, Becker & Hickl GmbH, Berlin, Germany) to generate the DToF for each gate (acquisition time 1 s for each gate). A reconstitution algorithm was used to preprocess and incorporate nine DToF signals (for each gate) into one DToF (to make the raw data using TG similar to FR). This algorithm acquires the preprocessed TG measurements after removing the effects of *DCR* and the variation of illuminated power on each gate. The algorithm then compares all the counted photons in each time channel from all interfered gates and selects the smallest value at each time channel bin to improve the SNR. In [156], higher *DR* (10^5^), was demonstrated in the final DToF histogram (outcome of the reconstitution algorithm) in comparison to the DToF generated by FR measurements (10^3.5^). Hence, 2D images were reconstructed using an algorithm based on MLT which can calculate higher orders with TG DToF as a result of the high *DR*. In other words, by increasing the MLT order, more late photons from deep regions of the target will be involved in the image reconstruction. The main result from this prototype is the increased depth sensitivity for inclusions up to 31 mm for 5 mm SDD, but the accuracy of localization and size estimation needs to be enhanced, particularly for deeper inclusions. However, eliminating the effects of new emerging sources of errors in TG, such as the memory effect, is a challenging issue [159,160]. The memory effect occurs in TG measurements as a result of the noise caused by the photons that impinge the SPAD detector while it is being turned off.

In [161], 3D images for two cylindrical inclusions (8 mm diameter, 12 mm height, and 10 mm separation) embedded inside a liquid Intralipid phantom were produced. Measurements were taken using nine source detector pairs with two different SDDs of 10 mm and 15 mm. The deepest obtainable sensitivity was 25 mm using either SDDs. However, the inclusions were only separable when the SDD was 10 mm. On the other hand, it was found that the depth sensitivity decreased when the inclusions are not at the same depth, since the shallower inclusion is taken as a priority by the inverse modeling causing the deeper inclusion to be difficult to detect. Therefore, a deeper inclusion will not be detected even if it is within a detectable depth (having a deeper inclusion at 15 mm and 20 mm). The researchers recommended the use of dense source detector pairs to improve the spatial resolution and the depth sensitivity of this prototype [161]. 

Eventually, from the reviewed TG prototypes it can be clearly seen that the gating capability is powerful in reflection geometry measurements to improve the SNR by reducing the effect of *DCR*, *P_AP_*, and counting more useful late photons. In addition, an ultrafast transition time (200 ps) of the gating for TG SPAD modules will allow for distinguishing late photons from early photons even for null SDD (≤5 mm). Also, these SPAD modules can work at high repetition rates up to 50 MHz which is a typical range for TR-DOS measurements (synchronized to a time-gating window with a laser pulse for each 20 ns) [115]. Table 3 lists the features and components of the TG prototypes mentioned in this section.

## 7. Discussions and Research Challenges

At present, TR-DOS systems that were previously complex, bulky, and costly for decades have become simpler, smaller and more affordable because of massive developments in photon timing and counting, as well as picosecond pulsed light source technologies. The cost and size were reduced by more than two orders of magnitude, particularly for photon timers. For example, ICCDs and streak cameras cost tens of thousands of USD, whereas SPADs, SiPMs, and TDCs only cost hundreds of USD. From the reviewed prototypes, several issues, challenges, and advances according to the components in the prototypes, are now described.

For pulsed light sources, custom pulsed-diodes and VCSELs have represented a good option for any portable TR-DOI prototype, and custom pulsed-diodes were successfully demonstrated in a portable FR-TR-DOS prototype using SiPM [66]. Also, a VCSEL was used with SiPM detector to construct a FR-TR-DOS prototype. This prototype was evaluated successfully using BIP and MEDPHOT protocols [107].

Silicon detectors for photon timing can be implemented to have several desirable features such as large active area, high *PDE* in the NIR range, short *IRF_Detector_* (tens of ps range), high dynamic range, low noise sources (*P_AP_*, crosstalk, *DCR* and memory effect), high count rate, and short *T_DEAD_*. However, trade-offs play an important role for achieving desirable features, since improving a feature leads to degrading another feature in the following ways.

1. Having a large active area is one of the most desirable features for detectors. However, noise and *IRF_SPAD_* increase greatly with active area dimensions larger than 200 μm for each pixel. Therefore, the active area can be enlarged by using arrays of pixels for SPADs or SiPMs. Incorporating a dedicated TDC for each pixel increases the noise and reduces the FF which minimizes the total active area of the detector. To avoid this limitation, each TDC should be shared by multiple pixels to increase the FF of pixels, and subsequently enlarge the total active area. However, by having more pixels share one TDC, the acquisition time becomes longer.
2. Having a high *PDE* in the NIR range can be achieved by increasing the thickness of the layer of absorption, and two approaches were demonstrated; the first being the use of a thick front-side illuminated detector and the other being the use of a thick back-side illuminated detector [116,128]. However, the maximum thickness of the detectors is restricted by the increase of noise and *IRF_Detector_* when the thickness is increased. When *IRF_Detector_* exceeds a few hundred ps, the detectors compatibility with TR-DOI systems is compromised.
3. Timing resolution (*IRF_Detector_*) can be enhanced by minimizing the active area and the thickness of the layer of absorption, however this results in the lowering of *PDE* and SNR. This is because photons will transmit for short distances inside the detector which reduces the probability of detection.
4. Dynamic Range (*DR*) can be improved tremendously by using TG instead of FR SPADs, but the *IRF_Total_* of the TG-TR-DOI systems are usually wider than FR-TR-DOI systems. However, the design of fast-gating SPADs requires high efficiency quenching circuits to ensure the fast transition time (range of few tens of ps) for the rising edge of the window.
5. To have low noise, reducing the contribution of sources of noise such as (*P_AP_*, crosstalk, *DCR*, and memory effect in TG measurements) is required. Selecting a proper *T_DEAD_* can eliminate the effects of *P_AP_*, but the maximum count rate will be degraded significantly if *T_DEAD_* is longer than necessary. Avoiding large active detectors and monitoring the temperature are the best methods to reduce the contribution of *DCR* to the total noise in the measurements. In TG measurements, a new source of background noise called the memory effect appears as a result of the huge number of early photons that hit the active area of the SPAD while it is in the OFF state. Therefore, zero SDD is not preferable because it increases the memory effect and subsequently reduces the SNR for late photons.

Conventional TCSPC devices obstruct the ongoing trend of minimizing size and reducing cost for each detection channel in TR-DOI prototypes. Therefore, using a TDC is a good alternative for meeting the small size and low cost requirements, and leads to the possibility of building TR-DOI systems with dense source–detector pairs in the foreseeable future. Some challenges were highlighted regarding TDC design and particularly with incorporating TDCs and detectors on the same chip [37]. The lack of a general FoM for TDC designs to evaluate the validity of a specific application obstructs the performance comparisons between different architectures of TDCs [162]. Moreover, the absence of a proper method of modelling TDCs hinders researchers in the design of custom TDCs. Because a modelling method does not exist, researchers must design, fabricate, and test TDCs practically before determining whether the TDCs will be compatible with their specific TR-DOI systems.

TR-DOI prototypes usually suffer from some limitations that require more research to be solved.

1. The inverse problems for the DE of diffused light are ill-posed, and there is no unique solution to them. No standard inverse problem modeling and image reconstruction tools are available yet. Therefore, a custom-made full analysis of DToF histograms is needed for each built prototype to interpret the raw data (DToF) and derive the optical properties of the target. However, advanced features of the prototypes such as image reconstructions, localization of inclusion, and accurate recovery of the optical properties are time consuming processes if all points in the DTOF are involved in the inverse problem solving.
2. Image reconstruction using only a few points of the DToF for measurements in reflectance and transmittance geometry reduces the time of processing significantly. Moreover, parallel computation tools such as GPU can be exploited to generate DToF histograms at multiple detection points during the iterative forward problem solver and to reconstruct several slices in DOT in parallel to reconstruct 3D images. However, for each slice the inverse problem must be solved in serial. To date, a fast forward problem model that uses a GPU is available, and the approach of using only a few points was validated in simulation for transmittance measurements only [69,86,87]. Therefore, the integration of these two approaches would lead to the reconstruction of images at a much faster speed (within tens of seconds).
3. Focusing on the most useful parts of DToF histograms in reflectance geometry was proven to be an effective method in recovering the optical properties and detecting inclusions at different depths. Developing TG detectors that can collect re-emitted photons during multiple gate-windows (with flexible size and delays between the gates) simultaneously is a powerful approach which was demonstrated and reported for dual-gate measurements [163]. The timing of detected photons during the two gates was achieved using a commercial TCSPC module, and custom-made timing electronics and their performance was proven to be similar [163]. Therefore, the benefits of using a dual window (or more) and replacing expensive TCSPC modules needs further investigation.

The detection of multiple inclusions at different depths using reflectance measurements is a known limitation for TR-DOI systems. This limitation appears when there are two inclusions within the depth sensitivity range, because the shallower inclusion usually distracts the inverse modelling from detecting the deeper inclusion. More sophisticated inverse problem solvers are required for TR-DOI systems to enable the detection of all inclusions without being distracted by shallow inclusions.

## 8. Conclusions

In this article, we discussed how silicon solid state detectors have contributed in developing the field of TR-DOS and DOI into a new era of affordability, portability, and compactness. In the first section of this paper, we introduced the physical principles of diffuse optical spectroscopy in the biological window (600–1000 nm), and the TR-DOI prototypes categorized based on geometry and methods of illumination and detection. The second and third sections mentioned the main component of TR-DOI prototypes and parameters for evaluating the equipment and the entire prototype respectively. The advantages and limitations of TR-DOI prototypes were specified, and the main features of the designing of SPADs that overcome the low detection efficiency of silicon in the range of red and the near infrared light (600–1000 nm) are stated in the fourth section. Moreover, the differences between FR and TG detection of photons, and FR vs. TG TR-DOI systems are mentioned in Section 4 as well. In the fifth section, the published FR-TR-DOI prototypes were reviewed, and the superiority of SiPMs was clarified. On the other hand, in the sixth section, TG prototypes were discussed and the benefits of the fast gating SPADs are explained, with an emphasis on their better capabilities in detecting deep inclusions. The mentioned FR and TG prototypes were compared based on the parameters indicated in the third section. Moreover, the rising popularity of SPAD based TR-DOI systems as a result of the promising advances in SPAD technology are discussed, specifically the distinctive capabilities of the ultrafast time-gating SPADs in comparison with traditional FR measurements. The seventh section discusses the limitations and challenges and the expected future developments according to each component in TR-DOI systems. 

In addition, FR-TR-DOI would mainly benefit from the recent and ongoing developments of cheap SiPM detectors and arrays of SPADs which have a large active area, and particularly when TDC units are integrated on the same chip as the detector. This can lead to building multichannel FR-TR-DOI prototypes that are portable, compact, and easy to use for a wide range of applications with fast data acquisition time. These FR-TR-DOI prototypes can have very good performance for transmittance geometry measurements for functional imaging and monitoring.

On the other hand, developing on-chip TG array of SPADs and SiPM would help in realizing low cost, compact, multichannel TG-TR-DOI portable prototypes for reflectance geometry measurements. These prototypes are expected to have high performance in depth selection and spatial resolution, particularly for the imaging of multilayered turbid targets such as functional brain imaging for neonates and adults.

Based on discussion (in Section 7) of the recent development of equipment that can be utilized for TR-DOI prototypes, it is noticeable that the main drawbacks of traditional TR-DOI systems—such as size, cost, and complexity—have been almost eliminated. New generations of TR-DOI will mainly utilize affordable equipment for light illumination, photon timing, and more accurate and computationally efficient image reconstruction tools. This will lead to an intense rise of interest in using this new generation of TR-DOI systems in imaging applications over the next few years.

## Figures and Tables

**Table 1:** Main features of SPADs and SiPM detectors used in TR-DOI prototypes.

Detector Type	Front-Side Illuminated SPAD				Backside-Illuminated SPAD	Excelitas-SiPM C30742-11-050-T1	Hamamatsu S10362-11-050C
Name	PDM50	PDM100	PDM200	CMOS350	CMOS130	SiPM1	SiPM2
Fabrication Technology	Custom (Planar) silicon			HV 0.35 μm CMOS	3D 130 nm CMOS	NA	NA
Operation Mode(s)	Free-Running and Time-Gated			Free-Running	Free-Running	Free-Running	Free-Running
No. of Pixels	1	1	1	128 × 128	2 Arrays (1 × 400)	400	400
Dimension of Pixel(s)	50 μm diameter	100 μm diameter	200 μm diameter	3.2 × 3.2 mm^2^	11 × 11 μm^2^ per pixel	50 × 50 μm^2^ per pixel	50 × 50 μm^2^ per pixel
Total Active Area	(1.963 × 10^3^ μm^2^)	(7.854 × 10^3^ μm^2^)	(3.1416 × 10^4^ μm^2^)	0.6144 mm^2^	28 μm^2^ per pixel	1 mm^2^	1 mm^2^
Time-Jitter (ps)	30	31	35	NA	260	100	<300
DCR (cps) @ Room-Temp.	2 k	5 k	60 k	NA	35,000	100 K	400 K
Dead Time (ns)	77	77	80	100	NA	NA	NA
Max. Count Rate (Mcps)	13	13	13	0.1	NA	27.6	NA
P_AP_ (%)	1	1	2	NA	NA	NA	NA
FoM_T_ Timing (m/s)	3400 k	NA	NA	NA	NA	152 k	NA

**Table 2:** Components, main features, and performance of FR TR-DOI prototypes.

Reference		[138]	[116]	[58]	[65]	[66]	[147,148]
**Light Source**	Type	Pulsed-diode	Supercontinuum	Solid-state Ti:Sapphire	Supercontinuum fiber laser	Pulsed-diode	Supercontinuum fiber laser
	Model	BHLP-700	SuperK Extreme, NKT Photonics	Tsunami, Spectra-Physics, USA	NKT Photonics, UK	Custom-designed (gain switching)	SC450, NKT Photonics, UK
	Wavelength (nm)	780 nm	750 nm	780 and 830 nm	820 nm	830 and 670 nm	Broadband (600–1350 nm)
	Illuminated Power (mW)	3 mW. parallel lines	17 mW	NA	NA	2 mW	3 mW/mm^2^
	Repetition Rate (MHz)	80 MHz	40 MHz	80 MHz	40 MHz	40 MHz	40 MHz
	Pulse Width (FWHM)	<250 ps	20 ps	4 ps	26 ps	240 ps	6 ps
**Detector**	Names	CMOS350	CMOS130	PDM50	SiPM1	SiPM1	SiPM2 and InGaAs PMT
	PDE(@λ)	NA	PDP > 12% (650–800 nm)	17% (780 nm) 12% (830 nm)	10% (@800 nm)	20% @ 670 nm and 8% @ 830 nm	For SiPM2: 17% @ 700 nm and 9% @ 800 nm
**Photon Timing**	Type	32 TDCs On-chip	100 TDCs on chip	PoliMi TCSPC module	TCSPC board (SPC-130, Becker and Hickl)	TDC on 0.35 μm CMOS	TCSPC
	Temporal Resolution (ps)	97	49.7	30	8	40	NA
	Dead Time (ns)	NA	NA	200	100	120	NA
**Target**	Type	Homogenous liquid/water	Homogenous solid/silicon mimic newborn head	Solid cylinder	Homogeneous liquid phantom/water	7 Solid phantoms by epoxy resin	6 positions in human body In Vivo
	Scatter Material	Intralipid	NA	NA	Intralipid	Titanium dioxide particles (TiO_2_)	Human tissue
	Absorber	India ink	NA	NA	Black India ink	printer toner powder	Human tissue
	Inclusion	NA	NA	NA	Five volumes of PVC cylinders absorbers	NA	Bone prominence
**DOS**	Geometry (Refl.Trans.)	Refl.	Refl.	Trans. 7 detectors	Refl.	Refl.	Refl. and Trans.
	SDD (mm)	20	20 to 40 mm	60°, 100°, 140°, 180°, 220°, 260°, 300°	3 cm SDD	3 cm SDD	2.5 cm in Refl.
	IRF (ps)	NA	<270	Avg. = 55	260	<280	NA
	Data acquisition time	18 min for 3 rows of pixels	NA	≈5 min; 2 s for each 1 of 144 measurements	NA	Repeating 1 s measurement 10 times, for each DToF	NA
**DOI**	Algorithm	TR$\overset{\mathrm{FFT}}{\to }$FD→DE iterative Tikhonov regularization	NA	Early Photons Time of Arrival (EPTA)	MLT	Best fitting of DToF using DE	NA
	Depth Sensitivity	30 mm	NA	25 mm	25 mm	NA	NA
	Spatial Resolution	5 mm at 1 cm depth	NA	<1.7 mm lateral localization	<1.5 mm lateral localization	NA	NA

**Table 3:** Components, main features, and performance of TG TR-DOI prototypes.

Reference		[152]	[43,135]	[63]	[153]	[132,154]	[156]	[157]	[161]
**Light Source**	Type	Pulsed diode laser	Supercontinuum fiber laser						
	Model	(PDL, Picoquant)	SC450, NKT Photonics		NKT Photonics	SC500-6, NKT Photonics	NKT Photonics		
	Wavelengths	672 and 758 nm	750 nm	710 and 820 nm	690 nm	760 and 860 nm	820 nm		
	Illuminated Power (mW)	0.1	0.01 to 1	60	NA	32	NA	55	NA
	Repetition Rate (MHz)	50	40	40	20	40	40	40	40
	Pulse Width (FWHM)	NA	NA	NA	<100 ps	<100 ps	NA	26 ps	
	Illuminated Area	1 mm diameter	1 mm diameter	0.4 mm diameter	NA	NA	NA	0.4 mm diameter	
**Detector**	Name	PDM50	PDM100 and PDM200	PDM100	PDM100	PDM100	PDM100	PDM100	PDM100
	PDE (@λ)	37% (650 nm)	22% (750 nm)	28% (710 nm) 13% (820 nm)	30% (690 nm)	20% (760 nm) 9% (860 nm)	13% (820 nm)	13% (820 nm)	13% (820 nm)
**Photon Timing**	Type	NA	TCSPC board (SPC-130, Becker and Hickl)		TCSPC board (SPC-134, Becker and Hickl)	TCSPC board (SPC-150, Becker and Hickl)	TCSPC board (SPC-130, Becker and Hickl)		
	Temporal Resolution (ps)	NA	8	8	8	6.5	8	8	8
	Dead Time (ns)	NA	100	100	25	100	100	100	100
**Target**	Type	2 Phantoms: 1. Homogeneous solid (epoxy resin) 2. Heterogeneous liquid (water)	homogeneous liquidwater	In-vivo brain tasks activity	homogeneous liquid	Two phantoms 1. Homogeneous liquid water 2. In-vivo brain tasks activity	homogeneous liquid/water (3 references)		
	Scatter Material	1. TiO_2_ 2. Intralipid	Intralipid	NA	Intralipid	Intralipid	Intralipid (3 references)		
	Absorber	1. Black toner (homogeneous) 2. Ink (heterogeneous)	Indian Ink	NA	Indian ink	India ink	Black ink (3 references)		
	Inclusion	PVC cylinder volume = 1 cm^3^ at 8 and 18 mm depth	PVC cylinder volume = 1 cm^3^	HbO_2_ and HHb changes	PVC cylinder volume = 1 cm^3^	Phan.1. five volumes of PVC cylinders absorbers Phan.2. changes of HbO_2_ and HHb	Epoxy resin cylinder TiO_2_ black ink (3 references)		
**DOS**	Geometry (Refl.Trans.)	Reflectance geometry (all references)							
	SDD (mm)	2	2	6	1	5	15	5 and 15	10 and 15
	Gate-Width (ns)	0.5	0.5	0.5	6	6	5	5	5
	Delays	NA	0 to 2500 ps (500 ps steps)	NA	0 to 2000 ps (250 ps steps)	25 ps step	NA	100 ps steps, 5 mm SDD: (7 gates) 15 mm SDD: (9 gates)	6 gates
	IRF (ps)	90 (672 nm) & 220 (758 nm)	150 for PDM100 and 300 for PDM200	NA	NA	NA	NA	NA	NA
	DR	10^6^	5×10^7^	5 × 10^7^	10^4^	NA	5 × 10^5^	10^6^	10^6^
	Data acquisition time	15 measurements; each one (60 s)	NA	10 measurements; each one 60 s, 1 s to record each DToF	1 s to record each DToF	20 measurements; each one 96 s, 1 s to record each frame (1024 gated DToFs)	9 s for 9 gated DToFs, 1 s for each	7 s for 7 gated DToFs, 1 s for each	6 s for 6 gated DToFs, 1 s for each
**DOI**	Algorithm	NA	NA	NA	NA	NA	Mellin-Laplace transform (MLT)		
	Depth Sensitivity	18 mm	30 mm using PDM100	NA	NA	<2.5 cm	25 mm	31 mm	31 mm
	Spatial Resolution	2 mm for inclusion at 8 mm depth	NA	NA	NA	NA	NA	NA	12 mm @ (15 mm depth)

## List of Acronyms and Abbreviations

BIP	Basic Instrumental Performance protocol
BSI	Backside-Illuminated
CMOS	Complementary Metal-Oxide-Semiconductor
CW	Continuous Wave
DCR	Dark Count Rate
DE	Diffusion Equation
DOI	Diffuse Optical Imaging
DOS	Diffuse Optical Spectroscopy
DOT	Diffuse Optical Tomography
DR	Dynamic Range
DToF	Distribution Time of Flight
FD	Frequency Domain
FEM	Finite Element Method
FF	Fill-Factor
fNIRS	functional Near-Infrared Spectroscopy
FoM	Figures-of-Merit
FR	Free-Running
FSI	Front-Side Illumination
FWHM	Full-Width Half-Maximum
GPU	Graphics Processing Unit
HbO_2_	Oxy-Hemoglobin
HHb	Deoxy-Hemoglobin
ICCD	Intensified Charge Coupled Device camera
IRF	Instrument Response Function
MC	Monte Carlo
MCP-PMT	Micro-Channel Plates
MLT	Mellin-Laplace Transform
MPE	Maximum Permissible Exposure
NIRS	Near Infrared Spectroscopy
OT	Optical Topography
P_AP_	After-Pulsing
PDE	Photon Detection Efficiency
PDP	Photon Detection Probability
PMT	Photomultiplier Tubes
PTA	Photon Time of Arrival
PVC	Polyvinyl Chloride
RTE	Radiative Transfer Equation
SDD	Source–Detector Distance
SiPM	Silicon Photomultiplier
StO_2_	tissue Oxygen Saturation
SNR	Signal-to-Noise Ratio
SPAD	Single-Photon Avalanche Diode
TCSPC	Time-Correlated Single-Photon Counting
TDC	Time-to-Digital Converter
*T_DEAD_*	Dead-Time
TG	Time-Gated
tHb	total-Hemoglobin
TiO_2_	Titanium Dioxide
TR	Time-Resolved
VCSEL	Vertical-Cavity Surface-Emitting Laser
